# Bioprosthetic tricuspid valve endocarditis caused by Acinetobacter baumannii complex, a case report and brief review of the literature

**DOI:** 10.1186/s13019-015-0377-8

**Published:** 2015-11-04

**Authors:** Qiang Chen, Hua Cao, Heng Lu, Zhi-huang Qiu, Jia-jun He

**Affiliations:** Department of Cardiovascular Surgery, Union Hospital, Fujian Medical University, Xinquan Road 29#, Fuzhou, 350001 P. R. China

**Keywords:** Tricuspid valve, Endocarditis, Acinetobacter baumannii

## Abstract

**Background:**

Species of the genus *Acinetobacter* are Gram-negative and highly drug-resistant bacilli that normally reside on the skin, oropharynx, and perineum. Several previous studies have reported prosthetic valve endocarditis due to *A. baumannii* infection.

**Case presentation:**

Here we present a case of late endocarditis of a bioprosthetic tricuspid valve due to *A. baumannii* complex in a patient who had surgical replacement with a bioprosthetic tricuspid valve six years previously.

**Conclusions:**

We completed tricuspid valve replacement using a 29 mm St. June mechanical prosthetic valve for the patient. Postoperatively, she received intravenous cefoperazone sodium and sulbactam sodium for 2 months and had good recovery.

## Background

Tricuspid valve infective endocarditis is an infrequent diagnosis, accounting for only 5 to 10 % of all cases of infective endocarditis, and is most common in intravenous drug users [1, 2]. Prosthetic valve endocarditis is a serious condition because it can result in death. Late prosthetic infection resembles other forms of infective endocarditis, both clinically and bacteriologically [3]. Here, we report a case of late infective bioprosthetic tricuspid valve endocarditis caused by *Acinetobacter baumannii*, with large vegetations and damage of the artificial valve. Although medical therapy was effective, replacement with a new mechanical tricuspid valve allowed the patient to achieve satisfactory recovery.

## Case presentation

A 56-year-old female was admitted to our hospital with a fever, double lower extremity edema, and recent weight loss. Eleven years ago, she had a mitral valve replacement with a 27 mm St. Jude mechanical valve, and six years ago she had a bioprosthetic tricuspid valve replacement with a 29 mm Edwards bioprosthetic valve due to severe tricuspid valve regurgitation. Since earlier this year, she had recurrent abdominal distension and double lower extremity edema, symptoms that were alleviated by treatment with a diuretic. She first presented to a local hospital in January with complaints of fever and cough for the previous month. A chest X-ray at that time indicated pulmonary infection, and transthoracic echocardiography showed bioprosthetic tricuspid valve calcification and medium regurgitation, but no vegetation. She was prescribed oral antibiotics and diuretics for two months. Despite decreased coughing and evidence of improvement in her chest X-ray films, she continued to suffer from abdominal distension and double lower extremity edema. Three different blood cultures were negative, and she was admitted to our hospital in Iune. The patient had no history of intravenous drug abuse or diabetes mellitus. Her body weight had declined by 10 kg since this year.

On admission to our unit, she had a temperature of 37 °C, atrial fibrillation with an average rate of 70 beats/min, and blood pressure of 120/60 mmHg. Auscultation indicated a diastolic sound below the xiphoid process. Laboratory tests showed leukocytosis (15.5 × 10^9^ WBCs/L) and anemia (75 g Hb/L). Her serum level of C-reactive protein was 15 mg/dL, erythrocyte sedimentation rate was 25 mm/h, and a chest X-ray film demonstrated scattered patches in both lower lung fields. The patient’s status began to deteriorate on the second day after admission. She suffered from intermittent whole-body chills and a fever spiking up to 39 °C. Transthoracic echocardiography showed large vegetations attached to the bioprosthetic tricuspid valve, damage to two of the three leaves, and severe tricuspid regurgitation. Blood cultures were prepared from three samples collected at hourly intervals, and led to a diagnosis of *A. baumannii* of the bioprosthetic tricuspid valve. Antimicrobial susceptibility testing showed that the isolate was sensitive to cefoperazone sodium and sulbactam sodium, but resistant to imipenem, penicillin, ampicillin, clindamycin, and linezolid. Thus, we initiated treatment with intravenous cefoperazone sodium and sulbactam sodium. After 4 weeks of antibiotic therapy, her pyrexia was completely controlled and intermittent blood cultures showed negative results. We therefore performed re-tricuspid valve replacement.

**Fig. 1:**
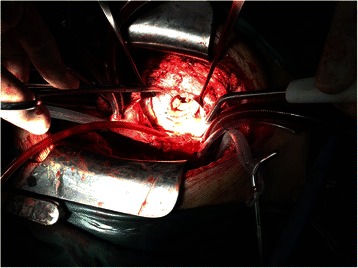
The damaged bioprosthetic tricuspid valve with vegetations

**Fig. 2:**
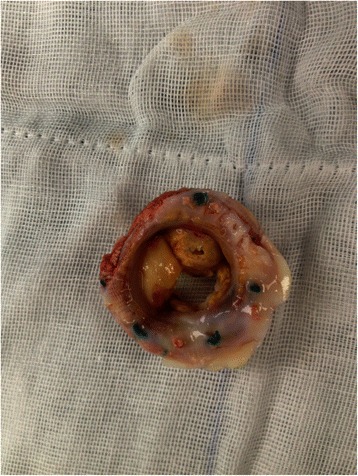
The damaged bioprosthetic tricuspid valve with vegetations

We completed valve replacement in the beating heart under cardiopulmonary bypass, during which we noted vegetations on all 3 leaves and damage to 2 of the leaves (Figs. 1 and 2). We used a dilute iodine solution in normal saline- cefoperazone sodium to irrigate the surface of the tricuspid annulus after removal of the bioprosthetic valve. A 29 mm St. June mechanical prosthetic valve was inserted according to the patient’s choice. Intraoperative cultures of the vegetations were negative. Postoperatively, she received intravenous cefoperazone sodium and sulbactam sodium for 2 months. Before discharge, all post-operative numerous blood cultures showed negative results and the patient reported that cardiac insufficiency-related symptoms were significantly ameliorated. During the follow-up period, there was no fever, no symptoms of infection, and no cardiac insufficiency.

*A. baumannii* was initially thought to be an organism of questionable pathogenicity, but has emerged as a major cause of nosocomial infections. Isolates of this species are frequently resistant to multiple antimicrobial agents, and can therefore cause life-threatening infections in susceptible patients [4–6]. Invasive medical procedures and use of broad-spectrum antibiotics increase the risk for *A. baumannii* infection. Consequently, episodes of *A. baumannii* bacteremia occur most frequently in patients admitted to intensive care units. Other reports indicate that the most common pathogens responsible for prosthetic valve endocarditis are of nosocomial origin, such as coagulase-negative staphylococci, *Staphylococcus aureus*, or Gram-negative bacilli [7]. Prosthetic valve endocarditis due to *Acinetobacter* is rare, and the present case of late bioprosthetic prosthetic valve endocarditis by *A. baumannii* is very unusual.

Olut and colleagues presented a case of early prosthetic valve endocarditis due to *A. baumannii* that was accompanied by a cutaneous eruption. In this case, a 6–7 mm vegetation was present on the aortic valve. Although appropriate antibiotics were started immediately, the patient died of septic shock and disseminated intravascular coagulation [8]. Menon and colleagues reported a case of infective endocarditis caused by *A. baumannii* complex in a 27-year-old male who underwent surgical repair of a ruptured aneurysm of the sinus of Valsalva one month previously. This patient died of sepsis before appropriate antibiotic therapy could be started [9]. Kumar et al. reported a 23-year-old female who underwent surgical replacement of the mitral valve and developed late endocarditis of the mechanical prosthetic valve due to *A. baumannii* complex. This patient, who received surgical replacement with a Starr-Edwards mechanical prosthetic valve 5 years previously, was treated with ofloxacin and amikacin and was soon afebrile [10]. Gradon et al. reported community-acquired infective endocarditis of a native valve that was caused by *A. calcoaceticus* subspecies anitratus. They further reported that 5 of 15 previously described patients with native valve endocarditis and 1 of 6 with prosthetic valve endocarditis died. These authors recommended therapy with antimicrobial agents known to be active against *Acinetobacter* [11].

In our patient, bioprosthetic tricuspid valve endocarditis developed six years after surgery, similar to the patient reported by Kumar et al. Although our patient’s symptoms and signs of cardiac insufficiency were initially misinterpreted, diagnosis was firmly established following isolation of *A. baumannii* from blood cultures. After admission, transthoracic echocardiography confirmed the presence of large vegetations attached to bioprosthetic tricuspid valve, which were not noted in her initial visit to a local hospital. Generally, imipenem is active against *A. baumannii*, but this species has developed resistance to multiple antimicrobial agents, such as imipenem, penicillin, ampicillin, clindamycin, and linezolid. Fortunately, the usual treatment for this patient is an active β-lactam alone, preferably one with a limited spectrum. Thus, we administered intravenous cefoperazone sodium and sulbactam sodium for about two months, a treatment that was successful.

The choice of the type of tricuspid prosthesis remains a matter of debate. In our patient’s second operation, a biological prosthetic valve was used in anticipation that this valve would have a slower degenerative evolution for the lower pressure stress in the right heart and was less likely to cause a thrombotic event [12–14]. There is no evidence that biological prosthetic valves are more likely to cause endocarditis. However, based on echocardiography, large vegetations were attached to the biological prosthetic tricuspid valve that damaged the biological leaves and led to severe tricuspid regurgitation. This necessitated surgery and valve replacement and the patient chose a mechanical valve for this third operation. Similar to many other reports on the outcomes following surgery for tricuspid valve infective endocarditis, we obtained a favorable result [15, 16]. Following surgery and intravenous antibiotic therapy, the patient’s symptoms of cardiac insufficiency had resolved.

## Conclusions

In conclusion, this case highlights a rare case of late infective endocarditis of a bioprosthetic tricuspid valve due to *A. baumannii* that was resistant to multiple antibiotics. Surgical replacement with a mechanical prosthetic valve accompanied by appropriate antibiotic treatment was successful.

Written informed consent was obtained from the patient for publication of this case report and any accompanying images. A copy of the written consent is available for review by the Editor-in-Chief of this journal.
